# Lamivudine plus adefovir combination therapy versus entecavir monotherapy for lamivudine-resistant chronic hepatitis B: a systematic review and meta-analysis

**DOI:** 10.1186/1743-422X-8-393

**Published:** 2011-08-08

**Authors:** Yun-Jian Sheng, Jun-Ying Liu, Shi-Wen Tong, Huai-Dong Hu, Da-Zhi Zhang, Peng Hu, Hong Ren

**Affiliations:** 1 Department of Infectious Diseases, Institute for Viral Hepatitis, Key Laboratory of Molecular Biology for Infectious Diseases, Ministry of Education, The Second Affiliated Hospital of Chongqing Medical University, Chongqing, China

**Keywords:** Chronic Hepatitis B, Lamivudine, Adefovir, Entecavir, Resistance, Combination therapy

## Abstract

**Background:**

Chronic hepatitis B virus (HBV) infection represents a serious global health problem and resistance to lamivudine (LAM) has become a serious clinical challenge. Previous rescue therapy for the treatment of chronic LAM-resistant hepatitis B infected patients included switching to entecavir (ETV) and adding adefovir (ADV) or tenofovir (TFV). At present, switching to ETV is not recommended for rescue therapy for LAM-resistant chronic hepatitis B (CHB). The aim of this report was to determine whether add-on ADV was a superior rescue strategy in the treatment of CHB patients with LAM resistance.

**Methods:**

We searched Medline/PubMed, EMBASE, Web of Knowledge, and the Cochrane Library. Relative risks (RRs) of virologic response, virologic breakthrough, normalization of serum alanine aminotransferase (ALT) levels and HBeAg seroconversion rates were studied. Factors predicting virologic response, standardized mean differences (SMD) in HBV DNA levels and safety were reviewed.

**Results:**

Six eligible trials (451 patients in total) were included in the analysis. The rate of virologic breakthrough in the ETV group was higher than that in the LAM plus ADV group. There were no statistical differences in virologic response, ALT normalization and HBeAg seroconversion in either group 48 weeks post treatment. LAM plus ADV combination therapy produced faster and greater HBV DNA reduction rates 24 weeks post therapy compared to ETV monotherapy. HBV DNA baseline levels and the initial virologic response (IVR) were predictive of the virologic response. Additionally, combination therapy or monotherapy were both well tolerated.

**Conclusions:**

LAM plus ADV combination therapy was more effective and produced longer-lasting effects than switching to ETV monotherapy in treating CHB patients with LAM resistance. However, considering the practical benefits and limitations of ADV, individualized therapy will be needed in patients with prior history of LAM resistant infections.

## Background

Chronic hepatitis B virus infection (HBV) poses a serious global health problem based on the approximately 350 million individuals suffering from chronic hepatitis B (CHB) infection worldwide [1]. Approximately 1 million patients die of liver failure, cirrhosis and hepatocellular carcinoma (HCC) as a result of HBV infection each year [2]. Studies have demonstrated that the risk of liver disease progression in patients with CHB is associated with elevated HBV DNA levels [3,4]. Therefore, the goal of therapy for CHB patients is to delay or prevent progression of liver disease by suppressing long-term HBV DNA replication [5]. Currently available antiviral drugs include interferon-alpha (INFα) and nucleos(t)ide analogue(NA) polymerase inhibitors (lamivudine, adefovir, entecavir, telbivudine and tenofovir). Treatments include individualized single-agent or combination therapies. Evidence-based medicine has demonstrated that effective antiviral treatment of CHB reduced the risk of long-term complications and improved patient survival [6,7]. Lamivudine (LAM) was the first nucleoside analog inhibitor to be approved for treatment of CHB infection and has been used widely in the treatment of CHB patients. However, a major shortcoming of LAM-based therapies is the development of drug resistant strains that develop with increasing frequency with treatment duration. The rate of LAM resistance is 24% after 1 year and approximately 70% after 5 years [8]. Furthermore, LAM resistance leads to the attenuation of HBV suppression and even causes hepatitis flare ups, hepatic decompensation and increased mortality rates [9], thereby posing a serious clinical challenge.

Previous rescue therapy for CHB patients with LAM resistance included switching to entecavir (ETV) and adding adefovir (ADV) or tenofovir (TFV) [9]. ADV-based therapies have been shown to be efficacious and safe for up to 5 years in HBeAg positive and negative patients [10,11]. However, sequential ADV monotherapy used as a LAM rescue therapy for the treatment of LAM resistant strains may result in an increased rate of multidrug-resistant HBV isolates. As a rescue therapy, LAM plus ADV combination therapy is superior to ADV monotherapy, resulting in effective viral suppression and a reduced risk of developing genotypic resistance [12,13]. ETV is a more potent antiviral agent with a high genetic barrier and induces a significant decline in viral loads in both HBeAg-positive and -negative treatment-naïve patients [14,15]. Compared to ADV in the treatment of nucleoside-naïve HBeAg-positive patients, ETV led to a more rapid and significant decrease in HBV DNA [16]. As a rescue therapy for CHB patients with LAM resistance, ETV monotherapy resulted in continued viral suppression and biochemical and serologic responses; however, sequential ETV therapy resulted in a 5-year cumulative probability of genotypic ETV resistance of 51% [17]. The updated 2009 American Association for the Study of Liver Disease (AASLD) Guidelines and the European Association for the Study of the Liver (EASL) Guidelines indicated that ETV was not the optimal treatment option and recommended adding ADV to treat CHB patients with LAM resistance if TDF was not available [18,19].

Much less is known about the efficacy of LAM plus ADV combination therapy versus a switch to ETV monotherapy as a rescue for CHB patients resistant to LAM. Recently, a number of studies have suggested differences in the efficacy between combination therapy and monotherapy since there are no evidence-based conclusive results. The purpose of the study described here was to systematically review and meta-analyze all published drug-based studies designed to treat CHB patients with LAM resistance and the use of LAM add-on ADV combination therapy (or ETV monotherapy) for the treatment of disease.

## Methods

### Literature search

Medline/PubMed, EMBASE, Web of Knowledge and the Cochrane Library were searched for relevant articles through March 24^th^, 2011 without language limitation. The search was designed using the key words "adefovir", "lamivudine", "entecavir", "lamivudine resistance", "lamivudine refractory" or "lamivudine failure". We obtained full articles and abstracts for all potentially relevant trials and the reference lists from retrieved documents were also searched. To maximize data requisition, we contacted authors whose articles contained insufficient information.

### Inclusion and exclusion criteria

The following inclusion criteria were used: (1) a randomized control trial-, cohort- or case-control-based study designs, (2) study population consisting of CHB patients with LAM resistance, and (3) intervention therapies consisting of LAM plus ADV versus ETV monotherapy. Patients were excluded if they (1) were not adults, (2) were pregnant, (3) infected with HBV strains resistant to drugs other than LAM, (4) co-infected with either hepatitis C, hepatitis D virus or human immunodeficiency virus (HIV), (5) presented with serious concurrent medical illness including concomitant renal failure, evidence of HCC or organ transplantation history, or (6) use of immunomodulatory drugs within the preceding 6 months. Data were not included in the meta-analysis if we could not gain sufficient statistical information.

### Efficacy measures

The primary efficacy end point was virologic response, defined as the proportion of patients with undetectable HBV DNA levels. Secondary end points included mean reduction of HBV DNA levels; virologic breakthrough, defined as an increase in serum HBV DNA by ≥ 1 log_10 _IU/ml or 1 log_10 _copies/ml above the nadir on treatment and biochemical response, defined as normalization of serum alanine aminotransferase (ALT) levels, HBeAg seroconversion, factors predicting virologic response and a drug safety evaluation designed to capture occurrence of adverse events.

### Data extraction

Two authors (Yun-Jian Sheng and Jun-Ying Liu) independently extracted the data using a pre-designed data extraction form. The following data were recorded: (1) the number of patients in respective studies, (2) details of the study design, (3) patient characteristics, (4) treatment doses and duration and (5) outcome measures performed as described earlier. Discrepancies were resolved with the assistance of an arbiter (Peng Hu) when necessary.

### Study quality

The quality of each study was assessed based on following criteria: (1) RCTs were assessed using the QUOROM guidelines as well as using the Jadad scale [20], (2) cohort and case-control studies had to meet the case matched by the patient's characteristics, (3) the studies selected had to have well-defined inclusion and exclusion criteria for patients and clear definitions of treatment responses. Discrepancies were resolved with the assistance of an arbiter (Peng Hu) when necessary.

### Statistical analysis

Outcomes were analyzed on an intent-to-treat basis. Meta-analysis was performed using fixed effect or random-effect methods, depending on the absence or presence of significant heterogeneity. Statistical heterogeneity between trials was evaluated by the chi-square and I-square (I^2^) tests, with significance set at *P *< 0.10. In the absence of statistically significant heterogeneity, the fixed-effect method was used to combine the results. When heterogeneity was confirmed (*P *< 0.10), the random-effect method was used. We used the relative risk (RR) of the main dichotomous outcomes as the measure of efficacy. The 95% confidence interval (CI) for the combined RR was also provided. Continuous outcomes were presented as a standardized mean difference (SMD) because one study reported a mean HBV DNA reduction using log_10 _IU/ml and other studies used log_10 _copies/ml [21-23]. The overall effect was tested using z scores calculated by Fisher's z transformation, with significance set at *P *< 0.05. Data analysis was carried out with the use of Review Manager Software 5.0 (Cochrane Collaboration, Oxford, United Kingdom).

## Results

### Search results and characteristics

**Figure 1:**
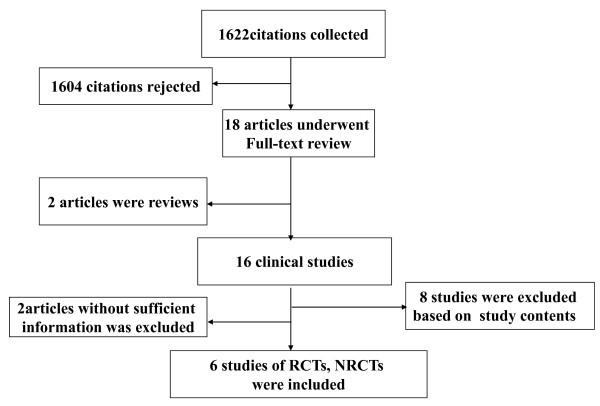
**Map of the literature search and selection process**.

We identified 1,622 citations *via *electronic searches, from which 6 were selected describing treatment of CHB involving 451 patients (225 treated with combination LAM plus ADV and 226 treated with ETV monotherapy) (Figure 1) [21-26]. Of the studies identified, three were RCTs [22,23,25]; one study [23] was published in Chinese and two published in English. Four studies [21-24] were published in full-text form and two [25,26] were published in abstract form. Because we did not gain sufficient statistical information from the data presented in abstract form, these two studies were not included in our analyses [27,28], and data presented in the Kim *et al*. study [24] (with the exception of virologic breakthrough) were not included in the meta-analysis. The respective studies utilized are summarized in Table 1.

**Table 1:** Characteristics of the included clinical trials in this study

Study	Location	Study design	Sample size (n)		Sex (M/F) (n)		Age (yrs)	HBeAg(+)/(-) (n)		LAM-resistance mutations at baseline (LAM+ADV) / ETV (n)	Regimen		Therapy period LAM+ADV *vs*. ETV	Baseline ALT (IU/L) LAM+ADV *vs*. ETV	HBVDNA level LAM+ADV *vs*. ETV	The detection limit of HBV DNA
			LAM +ADV	ETV	LAM +ADV	ETV	LAM+ADV *vs*. ETV	LAM +ADV	ETV		LAM+ADV	ETV				
Pellicelli 2009	Italy	RCTs	20	42	NA	NA	Mean (SD): 47 (11) *vs*. 48 (9)	NA (NS)		NA	LAM 100 mg/d + ADV 10 mg/d	1.0 mg/d	48 weeks *vs*. 48 weeks	NA	Mean (SD) (log_10 _IU/ml): 5 (1.0) *vs*. 4.63 (1.3)	NA
Qiu 2009	China	RCTs	30	30	NA	NA	NA	15/15	16/14	NA	LAM 100 mg/d + ADV 10 mg/d	1.0 mg/d	48 weeks *vs*. 48 weeks	Mean (SD): 141.1 (58.2) *vs*. 145.7 (61.9)	Mean (SD) (log_10 _copies/ml): 7.78 (2.62) *vs*.7.82 (2.32)	< 500 copies/ml
Ryu 2010	Korea	RCTs	47	45	34/13	38/7	Median (rang): 47 (20-68) *vs*. 41 (21-60)	39/8	42/3	M204I:14/15; L180M:1/0; M204I+M204V:0/1; M204I+L180M:8/9 M204V+L180M:14/13 M204I+M204V+L180M:10/7	LAM 100 mg/d + ADV 10 mg/d	1.0 mg/d	Median (rang), months: 12 (12-24) *vs*. 15 (12-27)	Median (range): 143 (26-1096) *vs*. 102 (17-677)	Median (rang) (log_10 _copies/ml): 7.61 (5.19-9.46) *vs*.7.10 (5.43-9.47)	< 300 copies/ml
Kim 2010	Korea	Cohort	36	24	25/11	21/3	Mean (SD): 46.8 (10.4) *vs*. 46.9 (8.7)	20/16	18/6	M204I or M204I+L180M: 24/11 M204V+L180M: 12/13	LAM 100 mg/d + ADV 10 mg/d	1.0 mg/d	24 months *vs*.24 months	Mean (SD): 227.6 (267.8) *vs*. 136.3 (134.2)	Mean (SD) (log_10 _copies/ml): 6.43 (1.40) *vs*. 6.51 (1.54)	< 300 copies/ml
Lee 2010	Korea	Cohort	48	33	NA (NS)	NA (NS)	NA (NS)	41/7	30/3	NA	LAM 100 mg/d + ADV 10 mg/d	1.0 mg/d	96 weeks *vs*. 96 weeks	NA (NS)	NA (NS)	< 140 copies/ml
Chung 2011	Korea	Cohort	44	52	35/9	33/19	Mean (SD): 53.7 (10.5) *vs*. 50.0 (10.4)	26/18	33/19	M204V+ L180M: 18/21 M204I+ L180M: 9/9 M204V/I+ L180M: 2/5 M204I: 14/17 M204V/I+ L180M+ V173L: 1/0	LAM 100 mg/d + ADV 10 mg/d	1.0 mg/d	48 weeks *vs*. 48 weeks	Mean (SD): 151 (125) *vs*. 193 (185)	Mean (SD) (log_10 _IU/ml): 6.86 (1.17) *vs*. 6.81 (1.03)	< 50 IU/ml

NA: not available; NS: not significant; iLAM: lamivudine; ADV: adefovir; ETV: entecavir; SD: Standard Deviation; RCTs: randomized controlled trials.

### Study quality

Quality assessment of the respective studies analyzed demonstrated that the RCTs had Jadad scores that ranged between1-5. Two full manuscripts [22,23] and one abstract [25] were RCTs and described withdrawn, but they did not describe the method of randomization in detail; these three studies received Jadad scores of 2. All trials had defined inclusion and exclusion criteria for patients and clear definitions of the treatment responses. In addition, all study populations had comparable baseline characteristics between the LAM plus ADV combination and ETV monotherapy groups.

### Virologic response

**Figure 2:**
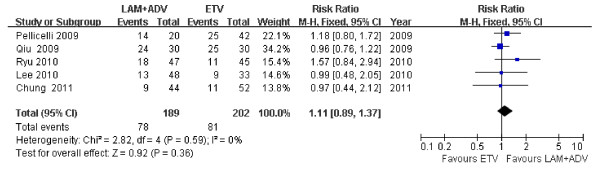
**Effect of LAM + ADV *vs*. ETV on virologic response 48 weeks post treatment**.

The rate of undetectable HBV DNA levels was 72.7% and 54.5% in both the ADV add-on LAM group and the ETV group 48 weeks post treatment in the Kim *et al*. study (*P *= 0.103) [24]. Analysis of the 5 trials included in the meta-analysis [21-23,25,26] showed undetectable HBV DNA levels in the LAM plus ADV group in 41.3% of patients compared to 40.1% of patients in the ETV group 48 weeks post treatment. Chi- and I square (I^2^) heterogeneity tests were assessed revealing no significant differences between treatment groups [Chi^2 ^= 2.82, df = 4 (*P *= 0.59); I^2 ^= 0%]; a summary estimate of the relative risk of LAM plus ADV versus ETV treatments using a fixed-effects approach. The rate of undetectable HBV DNA levels was not significant between groups [RR = 1.11, 95%CI (0.89, 1.37), *P *= 0.36] (Figure 2).

### Mean HBV DNA reduction levels

**Figure 3:**
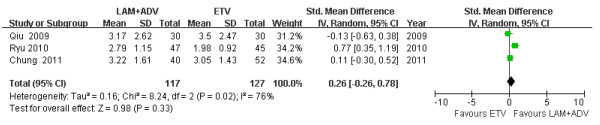
**Effect of LAM + ADV *vs*. ETV on the mean reduction of HBV DNA 12 weeks post treatment**.

**Figure 4:**
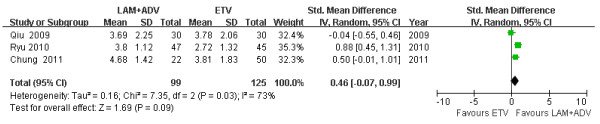
**Effect of LAM + ADV *vs*. ETV on the mean reduction of HBV DNA 48 weeks post treatment**.

**Figure 5:**
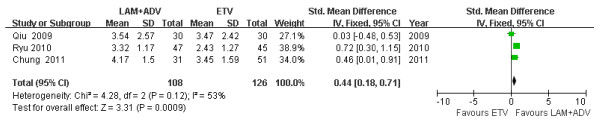
**Effect of LAM + ADV *vs*. ETV on the mean reduction of HBV DNA 24 weeks post treatment**.

Among three studies [21-23], chi- and I square (I^2^) analyses identified significant heterogeneity in HBV DNA levels between the treatment groups 12 [Tau2 = 0.16; Chi^2 ^= 8.24, df = 2 (*P *= 0.02); I^2 ^= 76%] and 48 weeks post treatment [Tau2 = 0.16; Chi^2 ^= 7.35, df = 2 (*P *= 0.03); I^2 ^= 73%]. A summary estimate of the SMD of the LAM plus ADV versus the ETV group using a random-effects approach identified a reduction in the HBV DNA SMD of 0.26 [ 95%CI (-0.26, 0.78), *P *= 0.33] (Figure 3) and 0.46 [ 95%CI (-0.07, 0.99), *P *= 0.09] (Figure 4) at 12 and 48 weeks, respectively. Heterogeneity was assessed and not found to be significant 24 weeks post treatment. A summary estimate of the SMD of LAM plus ADV versus ETV using a fixed-effects approach identified an SMD reduction of HBV DNA of 0.44 [95%CI (0.18, 0.71), *P *= 0.0009] (Figure 5). Furthermore, among three other studies not included in this meta-analysis, Lee *et al*. [26] found that the mean reduction in serum HBV DNA at 48 and 72 weeks post treatment was similar between the ETV and ADV combination therapy groups (-3.1 *vs*. -3.5 and -3.0 *vs*. -3.7 log_10 _copies/ml). However, 96 weeks post treatment, significant reductions were observed in the ADV combination group (-2.4 *vs*. -4.7 log_10 _copies/ml, *P *= 0.003). Kim *et al*. [24] reported that the ADV combination group had a significant reduction in HBV DNA levels compared to the ETV treatment group 24 weeks post treatment [-4.17 *vs*. -2.89 (*P *= 0.003) log_10 _copies/ml].

### Biochemical response

**Figure 6:**
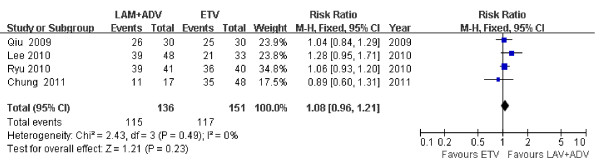
**Effect of LAM + ADV *vs*. ETV on ALT normalization 48 weeks post treatment**.

The ALT normalization rates were 74.3% and 75.0% for patients in the ADV add-on LAM group and ETV groups; respectively, 24 weeks post treatment in the Kim *et al*. study (P > 0.05) [24]. However, based on the chi-square and I-square (I^2^) analyses carried out in the meta-analysis, significant differences in ALT normalization rates were not detected [21-23,26] [Chi^2 ^= 2.43, df = 3 (*P *= 0.49); I^2 ^= 0%]; a summary estimate of the relative risk of LAM plus ADV versus ETV using a fixed-effects approach demonstrated that the rate of ALT normalization was not significant between groups 48 weeks post treatment [RR = 1.08, 95%CI (0.96, 1.21), *P *= 0.23] (Figure 6).

### Virologic breakthrough

**Figure 7:**
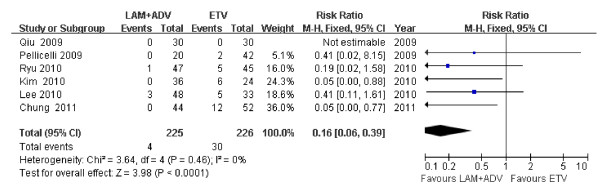
**Effect of LAM + ADV *vs*. ETV on virologic breakthrough 48 weeks post treatment**.

Based on the chi-square and I square (I^2^) analyses, significant differences in heterogeneity were not observed between treatment groups [Chi^2 ^= 3.64, df = 4 (*P *= 0.46); I^2 ^= 0%]; a summary estimate of the relative risk of LAM plus ADV versus ETV alone using a fixed-effects approach demonstrated that the rate of virologic breakthrough was higher in the ETV group 48 weeks post treatment [RR = 0.16, 95%CI (0.06, 0.39), *P *< 0.0001] (Figure 7).

### HBeAg seroconversion

**Figure 8:**
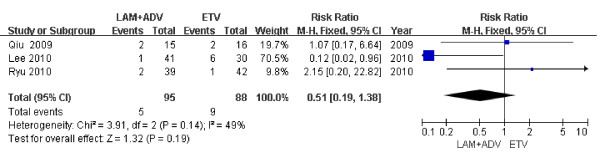
**Effect of LAM + ADV *vs*. ETV on HBeAg seroconversion 48 weeks post treatment**.

Only 3 studies examined in this analysis reported HBeAg seroconversion rates 48 weeks post treatment [21,24,27]. The rate of HBeAg seroconversion was higher in the LAM plus ADV combination therapy group than that observed for patients in the ETV group described by Kim *et al*. (38.9% *vs*. 0%), but no difference was observed in the Chung *et al*. study (31.3% *vs*. 68.8%, *P *= 0.229). Using the chi-square and I square (I^2^) analyses on the data examined for the meta-analysis [22,23,26] described here, significant differences in heterogeneity were not observed [Chi^2 ^= 3.91, df = 2 (*P *= 0.14); I^2 ^= 49%]; a summary estimate of the relative risk of LAM plus ADV versus ETV alone using a fixed-effects approach demonstrated that the rate of HBeAg seroconversion was not significant between groups 48 weeks post treatment [RR = 0.51, 95%CI (0.19, 1.38), *P *= 0.19] (Figure 8).

### Factors predicting the virologic response

Two studies described factors predictive of the virologic response in the different treatment groups [21,22]. Following multivariate analysis, independent parameters related to virologic responses were baseline ALT levels [odds ratio (OR), 1.003; 95% CI, (1.000, 1.006); *P *= 0.026] and baseline HBV DNA levels [OR, 0.495; 95% CI, (0.298, 0.823); *P *= 0.007] [22]. Another study demonstrated that baseline HBV DNA levels [OR, 0.304; 95% CI, (0.203, 0.457); *P *= 0.001] and the initial virologic response (IVR) [OR, 5.928; 95% CI, (2.880, 12.20); *P *= 0.001] were predictive of the virologic response [21]. Baseline HBV DNA levels were a critical parameter for predicting the virologic response and CHB patients with lower baseline HBV DNA levels presented with better virologic responses.

### Safety

Kim *et al*. [24] reported that 1 patient in the ETV monotherapy group experienced severe abdominal pain, nausea and diarrhea after 1 month of rescue treatment and treatment was therefore discontinued. One patient presented with elevated serum creatinine levels in the LAM plus ADV group. After modification of the ADV dose, serum creatinine levels declined. Another study reported that renal function was normal during the time of rescue treatment [23].

## Discussion

Over the past two decades, treatment of CHB has greatly improved with the availability of nucleos(t)ide analogs. The sustained suppression of serum HBV DNA to very low or undetectable levels has been associated with the prevention liver disease progression and inhibition of the development of long-term complications [6,7,29]. However, drug-resistance has been a serious clinical challenge to CHB treatment, especially for LAM-based therapies since it was the first widely used antiviral drug. Insufficient antiviral efficacy caused by drug resistance has resulted in attenuated viral suppression that may lead to significant clinical deterioration [9,30]. As a rescue therapy for LAM-resistant patients, previous treatment strategies have included add-on ADV and a switch to ETV or TFV [9]. However, ETV monotherapy in patients with LAM resistance is not currently recommended [18,19], although in some counties where TFV is not available or ADV is cost prohibitive, a switch to ETV is commonly carried out as a means of treating patients with LAM resistance. Currently, some studies have carried out direct comparisons between the antiviral efficacies of the respective rescue strategies. The aim of this analysis was arrive at an evidence-based conclusion based on available data regarding the efficacy of both rescue strategies.

Studies have shown that switching to ETV monotherapy for the treatment of CHB patients with LAM resistance was superior in maintaining viral suppression compared to continued LAM therapy [31,32]. However, LAM mutations conferring LAM resistance have previously been shown to result in reduced susceptibility to ETV *in vitro *[33] and ETV exerted positive selective pressure on LAM-resistant mutants *in vivo *[34]. Substitutions resulting in mutations at rtL180M/rtM204V in strains isolated from patients in the ETV group were treated as a consequence of LAM resistance may have resulted in the selection of strains with mutations at positions rtI169T, rtS202I/G, rtT184G or rtM250V. Substitutions at rtS202I/G, rtT184G and rtM205v were found in strains from patients in the ETV group in this study [21,24]. The cumulative probability of genotypic ETV resistance developing over 5 years was 51% in LAM resistant patients [17]. Studies also have shown that add-on ADV therapy for CHB patients with LAM resistance led to effective viral suppression [12,13,35] and patients receiving add-on ADV had a lower risk of developing genotypic resistance [12,13,35]. In our study, the rate of virologic breakthrough in the ETV group was higher than that in the LAM plus ADV group and Lee *et al*. [26] found that there was a significant reduction of HBV DNA in the LAM plus ADV group 96 weeks post treatment (*P *= 0.003). There were no statistical differences in the virologic response, ALT normalization and HBeAg seroconversion rates in either group 48 weeks post treatment. In previous studies [31,32,36,37], HBV DNA was undetectable 48 weeks post treatment in 21%-33.3% of patients after switching to ETV and undetectable in 35%-68% of patients after adding ADV to LAM treatment, which is inconsistent with data presented in this study. We speculated that differences in the HBV DNA detection limits, different definitions of baseline levels, regional genotypic difference in HBV isolates, the rate of HBeAg positive and the male to female ration may explain this difference. Furthermore, we found that LAM plus ADV combination therapy produced a more rapid and significant reduction in HBV DNA levels 24 weeks post treatment (*P *= 0.0009) compared to levels observed in patients receiving ETV monotherapy, even though there were no significant differences observed 48 weeks post treatment. Because higher HBV DNA loads represented a greater risk factor for the development of HCC and cirrhosis [3,4], the use of LAM plus ADV would be expected to result in a better clinical outcome than that observed for ETV only-treated CHB patients presenting with LAM resistance.

A new and emerging concept in the management of antiviral resistance is the superiority of add-on therapy rather than switching therapy as a means of preventing the development of subsequent multidrug resistant isolates [30]. The rtA181V/T and rtN236T substitutions have been identified as the primary ADV-resistance mutations [10]. Substitutions at rtA181 have been found after virologic breakthrough during LAM therapy in patients who were never treated with ADV. Two studies performed direct comparisons between LAM plus ADV combination therapy and ETV monotherapy in CHB patients resistant to both LAM and ADV [38,39]. Compared to CHB patients resistant to LAM alone in our study (regardless of combination therapy or monotherapy), the rate of virologic response was lower (16.7% *vs*. 41.3% in combination therapy, 12.3% *vs*. 40.1% in ETV monotherapy). Therefore, although the prevalent mutations at codon 181 were low (< 4% of patients with LAM resistance) [40-42], pretreatment resistance testing may be useful to fully characterize the viral variants present as a means of ensuring no cross-resistance between strains as a mean of optimizing drug regimens used in the treatment of CHB patients with LAM resistance.

Ryu *et al*. [22] showed insufficient virologic responses following ADV add-on LAM therapy in patients with higher baseline HBV DNA levels. The virologic response in patients with higher baseline HBV DNA levels (≥ 8 log_10 _copies/ml) was only 7.1% compared to 66.7% in patients with a lower baseline HBV DNA levels (5 ≤ HBVDNA < 6 log_10 _copies/ml) 48 weeks post treatment. Therefore, ADV add-on therapy may be more effective in treating LAM-resistant patients with lower baseline HBV DNA levels. In addition, add-on ADV should be implemented early because earlier addition of ADV at the time of virologic breakthrough (when the HBV DNA levels are low and before the development of biochemical breakthroughs) is associated with a significantly better long-term outcome in terms of HBV DNA suppression, ALT normalization and development of ADV-resistant HBV [35].

Ryu *et al*. further demonstrated that lower baseline HBV DNA levels and higher ALT levels were predictive of the virologic response [22] and Chung *et al*. [21] demonstrated that a lower viral load, ADV add-on therapy and IVR were independent predictors of favorable antiviral outcomes in LAM-resistant patients undergoing rescue therapy. Therefore, the present study suggested that the roadmap concept should incorporate baseline HBV DNA levels and IVR. Add-on ADV rescue therapy could be maintained for LAM-resistant patients with lower viral loads at baseline and IVR 24 weeks post antiviral treatment.

Combination therapy may have harmful effects. The potential for an increased risk of toxicity must always be considered when administering combination LAM plus ADV. In our study, most patients generally tolerated the drug regimens well; only one patient presented with elevated serum creatinine levels following treatment with LAM plus ADV, and after the ADV dose modification, serum creatinine levels declined. However, with the prolongation of treatment, 8.7% of patients presented with elevated creatinine levels (> 0.5 mg/dl) [11] and 16% of patients treated with LAM plus ADV combination therapy developed renal impairment and those with baseline GFR < 89 ml/min were at highest risk [43]. Short-term treatment was associated with fewer side effects following combination therapy in our study.

This systematic review carried out in this report had some limitations. First, some studies were not RCTs. Second, studies included in this systematic review were few and had small sample sizes. Third, studies published in abstract form (and some additional studies) did not report data that could be included in the meta-analysis; therefore, we could not carry out a deep analysis. Finally, the important limitation was publication bias. More high-quality, well-designed, randomized controlled, multi-center trails that are adequately powered will clearly be needed to guide evolving standards of care for treating CHB patients with LAM resistance.

In conclusion, LAM plus ADV combination therapy was more effective and longer lasting than switching to ETV monotherapy in the treatment of CHB patients with LAM resistance. However, considering the practical benefits and limitations of ADV therapy, individualized treatment regimens need to be implemented in treating patients with a history of prior LAM resistance.

## Abbreviations

HBV: Hepatitis B virus; HCC: Hepatocellular Carcinoma; CHB: Chronic Hepatitis B; ALT: alanine aminotransferase; AST: aspartate aminotransferase; RCTs: randomized controlled trials; NA: nucleos(t)ide analogues; IVR: initial virologic response; HIV: human immunodeficiency virus.

## Competing interests

The funding source had no influence on study design, in the collection, analysis, interpretation of the data, writing of the manuscript, or in the decision to submit the manuscript for publication. The contents are solely the responsibility of the authors and do not necessarily represent the views of the funding source.

## Authors' contributions

RH and HP conceived the study, provided funding supporting and revised the manuscript critically for important intellectual content. SYJ made substantial contributions to its design, acquisition, analysis and interpretation of data. LJY, TSW, HHD and ZDZ participated in the design, acquisition, analysis and interpretation of data. All authors read and approved the final manuscript.
